# i4mC-Deep: An Intelligent Predictor of N4-Methylcytosine Sites Using a Deep Learning Approach with Chemical Properties

**DOI:** 10.3390/genes12081117

**Published:** 2021-07-23

**Authors:** Waleed Alam, Hilal Tayara, Kil To Chong

**Affiliations:** 1 Department of Electronics and Information Engineering, Jeonbuk National University, Jeonju 54896, Korea; waleedtkr@jbnu.ac.kr; 2 School of International Engineering and Science, Jeonbuk National University, Jeonju 54896, Korea; 3 Advanced Electronics and Information Research Center, Jeonbuk National University, Jeonju 54896, Korea

**Keywords:** DNA methylation, regulate expression, CNN, deep learning

## Abstract

DNA is subject to epigenetic modification by the molecule N4-methylcytosine (4mC). N4-methylcytosine plays a crucial role in DNA repair and replication, protects host DNA from degradation, and regulates DNA expression. However, though current experimental techniques can identify 4mC sites, such techniques are expensive and laborious. Therefore, computational tools that can predict 4mC sites would be very useful for understanding the biological mechanism of this vital type of DNA modification. Conventional machine-learning-based methods rely on hand-crafted features, but the new method saves time and computational cost by making use of learned features instead. In this study, we propose i4mC-Deep, an intelligent predictor based on a convolutional neural network (CNN) that predicts 4mC modification sites in DNA samples. The CNN is capable of automatically extracting important features from input samples during training. Nucleotide chemical properties and nucleotide density, which together represent a DNA sequence, act as CNN input data. The outcome of the proposed method outperforms several state-of-the-art predictors. When i4mC-Deep was used to analyze *G. subterruneus* DNA, the accuracy of the results was improved by 3.9% and MCC increased by 10.5% compared to a conventional predictor.

## 1. Introduction

In DNA modification, methylation is a fundamental epigenetic tag that plays a major role in biological processes such as genomic imprinting, preservation of chromosomal stability, X-chromosome inactivation, cell cycle progression, and regulation of gene expression [1,2]. Cytosine methylation has been widely studied in both eukaryotic and prokaryotic genomes, where it creates bases such as 5-methylcytosine (5mC), 3-methylcytosine, and N4-methylcytosine [3,4]. 5mC is produced by adding a methyl group via DNA methyltransferase (DNMT) to the C5 position of cytosine, whereas 3-methylcytosine accrues due to the action of environmental alkylation agents [3,5]. 5mC is known to play a significant role in various biological functions [6,7] and is associated with diabetes, cancer, and neurological diseases [8,9,10]. In bacterial DNA, methylated DNA nucleobase 4mC is commonly explored, while the exact mechanisms and biological functions of 4mC modification sites are still limited [10].

The 4mC is relatively less investigated as compared to 5mC, and it has several roles such as correcting and controlling the DNA replication, gene expression levels, and cell cycle [2,11]. There are various experimental techniques for the identification of epigenetic cytosine nucleobases (4mC), which are namely, whole-genome bisulfite sequencing, reduced-representation bisulfite sequencing, mass spectrometry, transcription-activator-like effectors (TALEs) and single-molecule real-time sequencing (SMRT) [12,13,14]. Although these experimental techniques are sufficient for the identification of 4mC sites, they are expensive and laborious. Therefore, an efficient computational algorithm for the prediction of 4mC modification sites in large-scale genomic sequences would be greatly beneficial to the field. In the past decade, deep learning methods have achieved a remarkable performance in various fields such as image recognition [15,16,17], speech recognition [18], natural language processing [19] and bioinformatics [20,21,22,23,24].

Recently, several computational tools have been developed for the identification of 4mc sites, including iDNA4mC [25], 4mCPred [26], 4mCPred-SVM [27] and SOMM4mC [28]. All of these tools are based on machine learning techniques with hand-crafted features. iDNA4mC uses a support vector machine (SVM) with nucleotide chemical properties and nucleotide frequency as a feature vector for the detection of 4mC sites. 4mCPred and 4mCPred-SVM also use an SVM but have different mechanisms of feature representation. 4mCPred relies on two feature-encoding techniques, position-specific trinucleotide propensity (PSTNP) and electron–ion interaction pseudopotential EIIPs of trinucleotides, to encode the DNA sample as discrete value vectors. 4mCPredSVM applies four types of features for a combinatorial approach to 4mC site prediction, namely, K-mer dinucleotide frequency, mono-nucleotide binary encoding, dinucleotide binary encoding, and local position-specific dinucleotide frequency. SOMM4mC applies classical first and second-order Markov models to predict the 4mC epigenetic modification sites and shows better performance than the other previously mentioned tools. Furthermore, 4mCCNN [29] and DeepTorrent [30] are based on deep learning techniques. 4mCCNN utilizes one-hot encoding for data representation and convolution neural networks. DeepTorrent uses four type feature extraction techniques with convolution and LSTM layers. The previous deep learning model used complex architecture, which increases parameter and computational costs. Therefore, we need to design a more efficient model for 4mC site identification.

**Figure 1:**
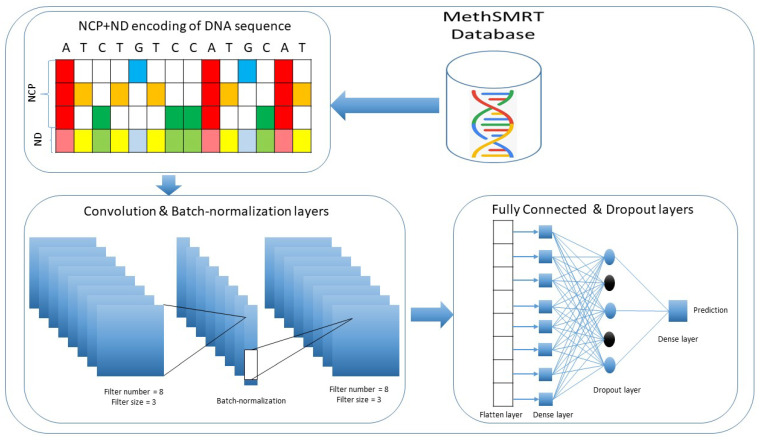
Demonstration of the data-flow and architecture of the proposed model.

In this study, we employ a convolutional neural network (CNN) to develop an accurate and efficient computational tool. The CNN is based on several layers, including a convolutional layer, batch normalization layer, flatten layer, dropout layer, and dense layer. The convolutional layer is used to automatically extract important features from an encoded DNA sequence. We apply the nucleotide chemical properties (NCP) and nucleotide density (ND) methods to encode the input DNA sequences [25,31,32]. Moreover, we use the batch normalization and dropout layers to control overfitting. Finally, we utilize the dense layer with sigmoid activation to classify the DNA sequence as either a 4mC site or a non-4mC site. We apply the 10-fold cross-validation technique with standard evaluation metrics in the field of bioinformatics [33,34,35] to evaluate i4mC-Deep. The outcomes of i4mC-Deep are superior to those of previous tools. The architecture of the i4mC-Deep has been illustrated in Figure 1. Finally, we have developed a free online web server to facilitate research in academia and industry, which is available at http://nsclbio.jbnu.ac.kr/tools/i4mC-Deep/, accessed on 15 July 2021, and we have provided the source code at: https://github.com/waleed551/i4mC-Deep, accessed on 15 July 2021.

## 2. Materials and Methods

This section includes the benchmark datasets, proposed model and evaluation measures.

### 2.1. Benchmark Dataset

The dataset plays a very important role in the development of an efficient and reliable computational tool. We utilized data from six different species of prokaryotes and eukaryotes, *Caenorhabditis elegans*, *Drosophila melanogaster*, *Arabidopsis thaliana*, *Escherichia coli*, *Geoalkalibacter subterraneus*, and *Geobacter pickeringii*. The datasets were constructed by [25] using the MethSMRT database [36]. The benchmark datasets contain 1554, 1769, 1978, 388, 906, and 569 positive and negative samples, respectively. Each sequence in the six datasets has a centrally located cytosine (C), with a length of 41 nt. The summary of six species benchmark datasets is shown in Table 1.

### 2.2. Deep Learning Approach

In this study, we used a convolutional neural network (CNN) to predict 4mC modification sites from DNA samples. The CNN is capable of automatically extracting important features from the input samples during training. The CNN input of DNA sequences is encoded by nucleotide chemical properties (NCP) and nucleotide density (ND). Each input DNA sequence has four different chemical properties that are derived from three groups based on the presence of hydrogen bonds, functional groups, and ring structures. In detail, during the formation of secondary structures, A and T form weak hydrogen bonds, whereas C and G form strong bonds; G and T contain a keto group, while A and C contain an amino group; and C and T have structures with only one ring, whereas A and G have two ring structures. Accordingly, the chemical properties of the four nucleotides can be represented in three coordinates (x, y, and z), and each coordinate can be assigned a value of 0 or 1. Thus, the four nucleotides that make up a DNA sequence can be represented in the Cartesian coordinate system. The resultant coordinates for A, C, G, and T are (1, 1, 1), (0, 0, 1), (1, 0, 0) and (0, 1, 0), respectively. The nucleotide density contains information on the frequency of each nucleotide in a given DNA sequence. Thus, we integrate NCP and ND into a 4-channel feature vector. The CNN has steps, or layers, including the convolution layers, activation layers, normalization layers, flatten layers, dropout layers, and fully connected layers. Several hyper-parameters are tuned during training, such as filter size, kernel size, strides, and dropout probability. The best hyper-parameter has been selected based on the validation loss. The gird search range of hyper-parameters is shown in Table 2. The optimal hyper-parameters for convolution layers are 2, the filter size is 8 for both layers, padding is the “same” for both layers, kernel size is 3 for both layers, and the dropout probability is 0.3. The mathematical representation of these layers is as follows:

(1) $$\mathrm{Conv}{\left(S\right)}_{ij}=\mathrm{ReLU}\left(\sum_{s=0}^{Z-1}\sum_{n=0}^{I-1}W_{sn}^{k}S_{j+s,n}\right)$$

(2) $$f=w_{d+1}\sum_{k=1}^{d}m_{k}w_{k}z_{k}$$

(3) $$\mathrm{ReLU}\left(x\right)=\left\{\begin{array}{c} x\;ifx>0\\ 0\;ifx\leq 0 \end{array}\right.$$

(4) $$\mathrm{Sigmoid}\left(x\right)=\frac{1}{1+e^{-x}}$$

We adopted several evaluation measures, namely, sensitivity, specificity, accuracy, and Mathew correlation coefficient (MCC), to enable a fair evaluation of the proposed tools. Both convolution layers and one dense layer are followed by a nonlinear function rectified linear unit (ReLU), while the last dense layer is followed by a sigmoid activation function that classifies the given DNA sequence as a 4mC or non-4mC site. The sigmoid activation function scales the output into the range [0, 1]. Moreover, we applied l2 regularization and dropout regularization to avoid overfitting from the network. The proposed model has been optimized using Adam with a learning rate of 0.001. The best batch size for the proposed model is 32, and the ideal number of epochs is 200 with early stopping. This bioinformatics tool is implemented in Python using the Keras framework.

### 2.3. Evaluation Measures

In this study, we applied four standard measures that are widely used in binary classification tasks to evaluate the performance of bioinformatics tools [37,38,39,40,41,42,43], namely, accuracy (ACC), sensitivity (SN), specificity (SP), and Matthew correlation coefficient (MCC). Mathematically, these measures are expressed as follows:

(5) $$\mathrm{ACC}=1-(\frac{N_{-}^{+}+N_{+}^{-}}{N^{+}+N^{-}})$$

(6) $$\mathrm{SN}=1-(\frac{N_{-}^{+}}{N^{+}})$$

(7) $$\mathrm{SP}=1-(\frac{N_{+}^{-}}{N^{-}})$$

(8) $$\mathrm{MCC}=\frac{1-(\frac{N_{-}^{+}+N_{+}^{-}}{N^{+}+N^{-}})}{\sqrt{\left(1+\frac{N_{+}^{-}-N_{-}^{+}}{N^{+}}\right)\left(1+\frac{N_{-}^{+}-N_{+}^{-}}{N^{-}}\right)}}$$

where $N^{+}$ represents methylcytosine sites, $N^{-}$ represents nonmethylcytosine sites, $N_{+}^{-}$ represents methylcytosine sites that are incorrectly identified as non-methylcytosine sites, and $N_{-}^{+}$ shows the number of non-methylcytosine sites that are predicted to be methylcytosine sties.

## 3. Result and Discussion

### 3.1. Comparison with Other State-of-the-Art Tools

**Figure 2:**
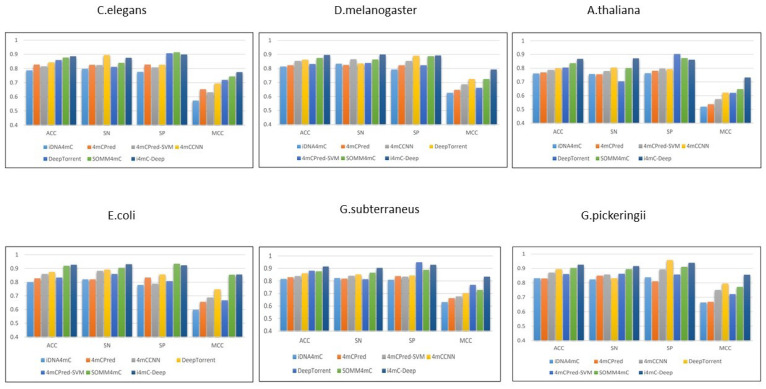
Shows the performance comparison of the proposed tool and other existing state-of-the-art tools.

**Figure 3:**
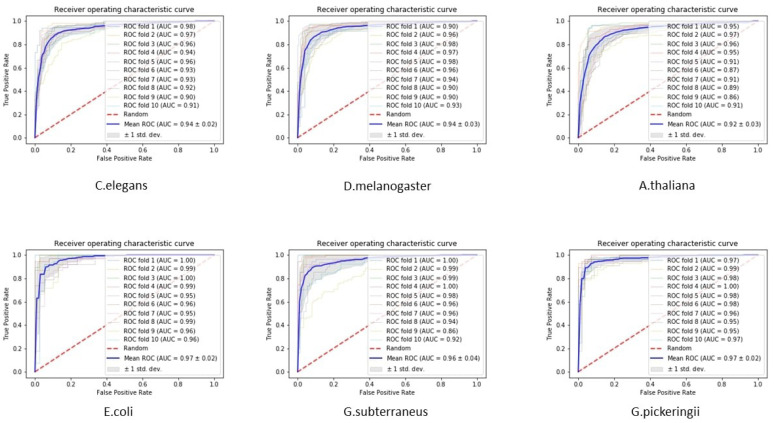
Demonstration of the test dataset receiver operation characteristic curve (ROC) of the ten folds and their standard deviation for six species.

Here, we compare the performance of the proposed method with other state-of-the-art tools, including iDNA4mC [25], 4mCPred [26], 4mCPred-SVM ([27]), and SOMM4mC ([28]). Table 3 and Figure 2 demonstrate the performance according to the four basic evaluation metrics of the proposed method and existing methods. Figure 3 shows the receiver operation characteristic curve (ROC) of six species along with standard deviation errors in ten folds. As done in previous studies, we also utilize 10-fold cross-validation and similar measurement parameters to enable a fair comparative evaluation. The results show that the accuracy of the proposed method is superior to that of existing state-of-the-art methods for all benchmark datasets. In detail, 4mC-Deep improved the classification of the *C. elegans* benchmark dataset by 1.0% accuracy, 3.5% sensitivity, and 3.1% MCC. In *D. melanogaster*, accuracy was improved by 2.1%, specificity by 0.6%, sensitivity by 3.6%, and MCC by 6.7%. In *A. thaliana*, accuracy was improved by 2.9%, sensitivity by 7.1%, and MCC by 8.4%. In *E. coli*, 4mC-Deep improved accuracy, sensitivity, and MCC by 0.8%, 1.9%, and 0.1% respectively. All measurement parameters were improved in *G. subterraneus*: 3.9% improvement in accuracy, 3.8% for specificity, 4.0% for sensitivity, and 10.5% for MCC. In *G. pinckeringii*, classification was improved by 2.3%, 2.7%, 2.0%, and 8.3% for accuracy, sensitivity, specificity, and MCC, respectively. As we notice, the specificity of the previous tools is higher than the proposed tool. Specificity demonstrates the correct detection of the true-negative classes, while sensitivity demonstrates the correct detection of the true-positive classes. Accordingly, the specificity and sensitivity are both highly required for better performance of the models. If we look at the performance of the previous method, the variance between the sensitivity and specificity is higher, which decreases the model accuracy and MCC. On the other hand, the performance of the proposed tool is much higher as compared with existing tools because the variance is less between sensitivity and specificity. Therefore, it is evident that the outcomes of our proposed 4mC-Deep tool outperformed all existing tools on six species benchmark datasets.

Finally, we compared the proposed model with our previous published tool DNA4mC-Deep [44], which was proposed for *F. vesca* and *R. chinensis*. We trained DNA4mC-Deep on the six species in this study. We found that the i4mC-Deep model performs better in almost all species. The comparison results of i4mC-Deep and the trained DNA4mC-Deep are given in Figure S8 and Table S1 in Supplementary File. Furthermore, we tested the pretrained cross-species model DNA4mC-Deep, and the results are given in Table S2 in Supplementary File.

### 3.2. Interpretation of the Proposed Tool

**Figure 4:**
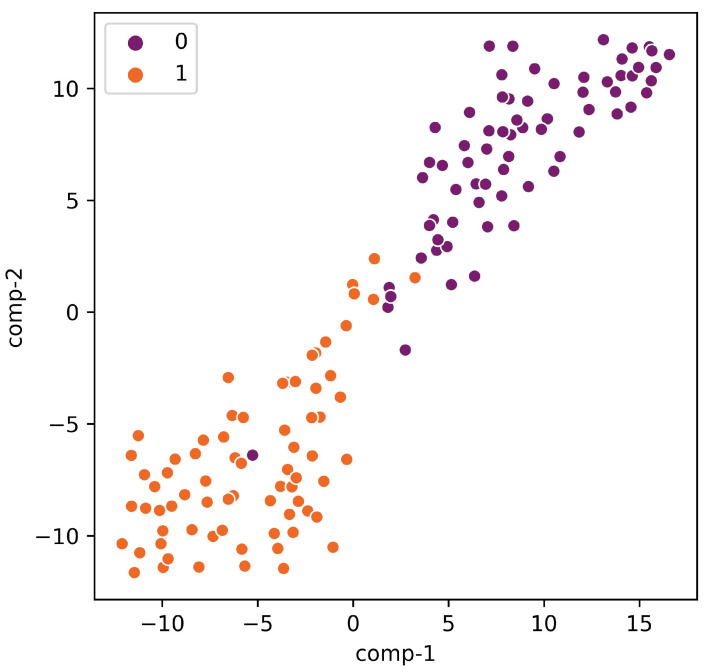
The t-SNE visualization of the learned features of the *G. subterraneus* dataset using the proposed model. The “0” represents the features of the negative samples and “1” represents the features of the positive samples.

The interpretation of the trained models provides the biologist insights for a better understanding of the task at hand. The developed models for the six species learned separable features. These separable features made the task of the classifier easier and helped in outperforming the previous methods. We extracted the learned features from each trained model of every species in the study from the flatten layer. This layer represents the learned features by the model during training. Then, we used t-distributed stochastic neighbor embedding (tsne) to visualize the learned features. For example, Figure 4 shows the learned features by the *G. subterraneus* model. It can be seen that the proposed model was able to learn separable features so the achieved performance is superior compared with the state-of-the-art models for the same dataset. The same behavior was obtained from the other models in our study, as shown in Figure S1 in Supplementary File.

Furthermore, we studied in silico mutagenesis using the trained models on the six species in our study. This method was applied in various studies [44,45,46] to interpret the effects of mutations using the trained deep learning model.

For every input sequence $s=(s_{0},s_{2},\ldots ,s_{40})$ we generated a 41×4 matrix by mutating each nucleotide at every position into the other nucleobase. For every in-silico mutation, we calculated the absolute prediction differences between the reference sequence and the mutated sequence.

**Figure 5:**
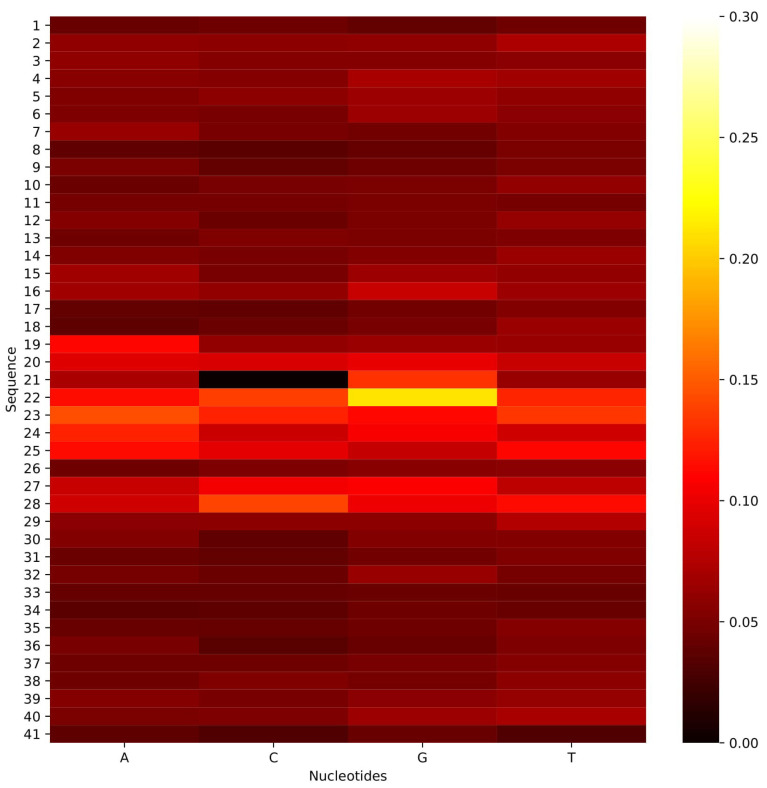
Demonstration of a heatmap visualization of in silico mutation of *G. subterraneus*.

The heat map of in silico mutation analysis is shown in Figure 5 for *G. subterraneus*, and Figure S2 in Supplementary File for the other species in our study. These heatmaps show that the mutation in the center of the sequence could have the highest impact on the prediction performance.

**Figure 6:**
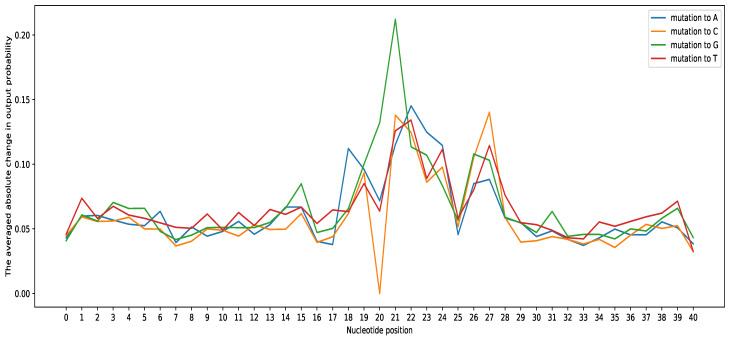
The effect of the mutations on the prediction probability in *G. subterraneus*.

To further analyze the results, Figure 6 shows the effects of mutation on the prediction result for *G. subterraneus* and other species in Supplementary file Figures S3–S7. It can be seen that mutations in the flanking regions, positions 0 to 17 and positions 28 to 40, have a small impact on the prediction performance. However, the mutations at positions 18 to 27 alter the prediction by more than 10%. The most noticeable alteration in the prediction occurs due to the mutation to Guanine (G) at position 21 of more than 20%.

## 4. Web-Server

**Figure 7:**
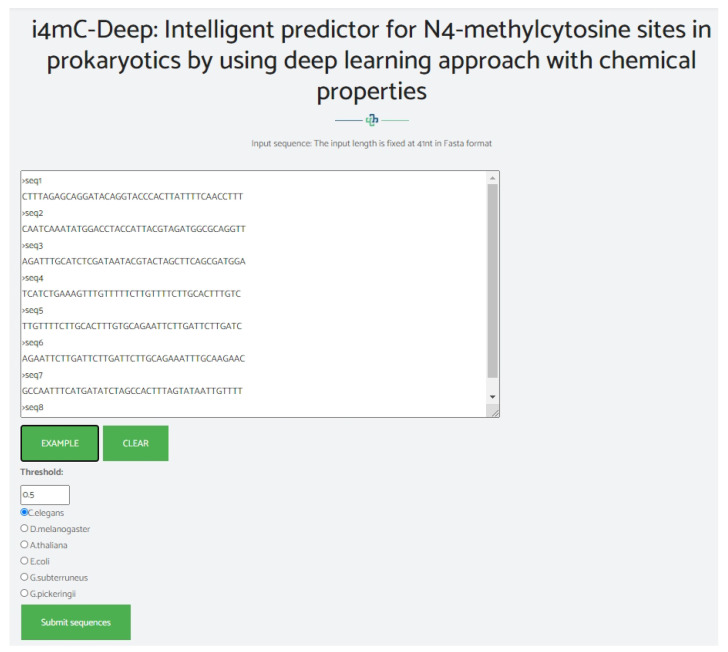
Demonstration of the web-server window where the users can put the DNA sequences in Fasta format directly for the prediction of 4mC site.

**Figure 8:**
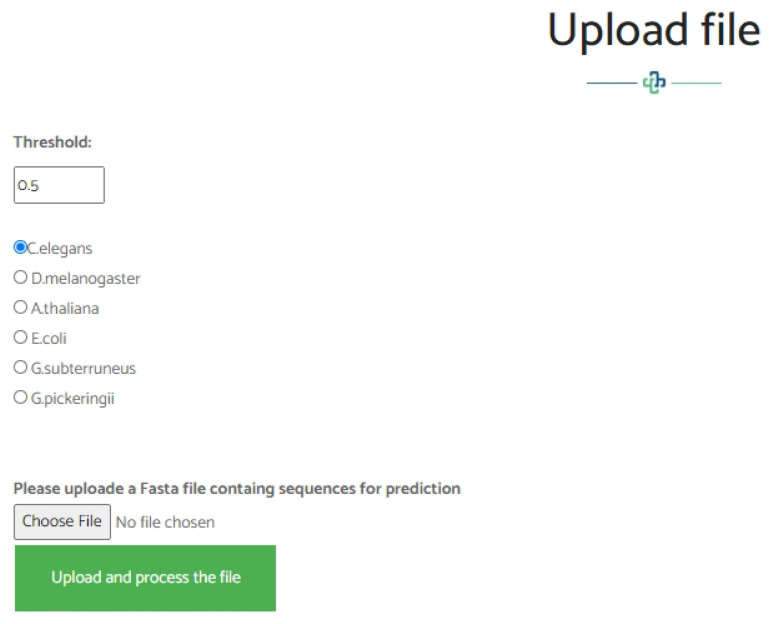
Demonstration of the web-server window where the users can upload the DNA sequence in the Fasta file.

We established a user-friendly and freely accessible web server for the proposed method to facilitate future studies. The established web server supports the classification of 4mC sites using either direct sequences in Fasta format, as shown in Figure 7, or direct upload of a Fasta file, as shown in Figure 8. The web server uses the Python programming language with the Flask library. It is available at http://nsclbio.jbnu.ac.kr/tools/i4mC-Deep/, accessed on 15 July 2021.

## 5. Conclusions

DNA N4-methylcytosine is an important biochemical modification that regulates gene expression. Therefore, an accurate and efficient computational tool, i4mC-Deep, was developed to identify 4mC sites in DNA sequences. i4mC-Deep has a layered architecture with a convolution layer, batch normalization layer, dropout layer, and dense layer. NCP and DN techniques are used to encode a DNA sequence to discrete values. The convolution layer automatically extracts features from a given input DNA sequence. Hyper-parameter searching is applied to identify the optimal parameter. The outcomes of four evaluation metrics demonstrate that i4mC-Deep is more reliable and efficient than comparable tools. The i4mCDeep tool will be invaluable for researchers in academia and industry. Finally, we developed a web server for the proposed method, which is freely accessible online at http://nsclbio.jbnu.ac.kr/tools/i4mC-Deep, accessed on 15 July 2021.

## Figures and Tables

**Table 1:** The summary of six species benchmark datasets.

Species	Sequences		Total
*C. elegans*	Positive	1554	3108
	Negative	1554	
*D. melanogaster*	Positive	1769	3538
	Negative	1769	
*A. thaliana*	Positive	1978	3956
	Negative	1978	
*E. coli*	Positive	388	776
	Negative	388	
*G. subterraneus*	Positive	906	1812
	Negative	906	
*G. pickeringii*	Positive	569	1138
	Negative	569	

**Table 2:** The ranges of the tuned hyper-parameters.

Hyper-Parameters	Range
Filters of Conv1D	[8,16,32]
Conv1D kernel size	[3,5,7]
Conv1D Strides	[2,3]
Dropout	[0.2,0.3,0.4,0.5]
Dense layer units	[8,16,32]

**Table 3:** The performance comparison between the i4mC-Deep and the existing computational tools for 4mC sites.

Datasets	Methods	ACC	SN	SP	MCC
*C. elegans*	iDNA4mC	0.786	0.797	0.775	0.572
	4mCPred	0.826	0.825	0.826	0.652
	4mCPred-SVM	0.815	0.824	0.807	0.631
	4mCCNN	0.842	0.894	0.825	0.694
	DeepTorrent	0.858	0.810	0.906	0.719
	SOMM4mC	0.876	0.839	**0.913**	0.743
	i4mC-Deep	**0.886**	**0.874**	0.898	**0.774**
*D. melanogaster*	iDNA4mC	0.812	0.833	0.791	0.625
	4mCPred	0.822	0.824	0.821	0.646
	4mCPred-SVM	0.830	0.838	0.822	0.661
	4mCCNN	0.853	0.864	0.853	0.686
	DeepTorrent	0.861	0.834	0.889	0.724
	SOMM4mC	0.874	0.862	0.886	0.724
	i4mC-Deep	**0.895**	**0.898**	**0.892**	**0.791**
*A. thaliana*	iDNA4mC	0.760	0.757	0.762	0.519
	4mCPred	0.768	0.755	0.780	0.536
	4mCPred-SVM	0.787	0.778	0.796	0.573
	4mCCNN	0.797	0.803	0.792	0.621
	DeepTorrent	0.803	0.703	**0.903**	0.620
	SOMM4mC	0.836	0.800	0.872	0.647
	i4mC-Deep	**0.865**	**0.871**	0.861	**0.731**
*E. coli*	iDNA4mC	0.799	0.820	0.778	0.598
	4mCPred	0.826	0.819	0.832	0.655
	4mCPred-SVM	0.833	0.858	0.807	0.666
	4mCCNN	0.859	0.881	0.788	0.687
	DeepTorrent	0.873	0.891	0.855	0.747
	SOMM4mC	0.918	0.903	**0.934**	0.853
	i4mC-Deep	**0.926**	**0.930**	0.922	**0.854**
*G. subterraneus*	iDNA4mC	0.815	0.822	0.808	0.630
	4mCPred	0.828	0.818	0.837	0.662
	4mCPred-SVM	0.837	0.840	0.834	0.674
	4mCCNN	0.860	0.851	0.843	0.703
	DeepTorrent	0.880	0.813	**0.948**	0.768
	SOMM4mC	0.876	0.864	0.888	0.728
	i4mC-Deep	**0.915**	**0.904**	0.926	**0.833**
*G. pinckeringii*	iDNA4mC	0.831	0.824	0.838	0.663
	4mCPred	0.830	0.850	0.810	0.668
	4mCPred-SVM	0.860	0.863	0.858	0.721
	4mCCNN	0.871	0.857	0.893	0.750
	DeepTorrent	0.894	0.831	**0.957**	0.795
	SOMM4mC	0.903	0.895	0.911	0.772
	i4mC-Deep	**0.926**	**0.915**	0.938	**0.855**

## Data Availability

No new data were generated in this study.

## Supplementary Materials

The following are available online at https://www.mdpi.com/article/10.3390/genes12081117/s1. Figure S1: The t-SNE visualization of the learned features for *C. elegans* (a), *A. thaliana* (b), *D. melanogaster* (c), *E. coli* (d), and *G. pickeringii* (e); Figure S2: Heatmaps of in silico mutagenesis analysis for *C. elegans* (a), *A. thaliana* (b), *D. melanogaster* (c), *E. coli* (d), and *G. pickeringii* (e); Figure S3: The effects of mutation on the prediction result in *A. thaliana*; Figure S4: The effects of mutation on the prediction result in *C. elegans*; Figure S5: The effects of mutation on the prediction result in *D. melanogaster*; Figure S6: The effects of mutation on the prediction result in *E. coli*; Figure S7: The effects of mutation on the prediction result in *G. pickeringii*; Figure S8: The comparison results between i4mC-Deep and DNA4mC-Deep after training the DNA4mC-Deep model on the six species in this study; Table S1: The performance comparison between the i4mC-Deep and the DNA4mC-Deep after training the DNA4mC-Deep model on six species; Table S2: The performance comparison between i4mC-Deep and pretrained cross-species model DNA4mC-Deep.
