# Supplementary material for: i4mC-Deep: An Intelligent Predictor of N4-Methylcytosine Sites Using a Deep Learning Approach with Chemical Properties
Source: Genes (Basel). 2021 Jul 23;12(8):1117. doi: 10.3390/genes12081117 (PMC8393747; doi:10.3390/genes12081117)
Supplement: Supplementary file 1 [file genes-12-01117-s001.zip › genes-1270927-supplementary.pdf]

Supplementary File

i4mC-Deep: An intelligent predictor of N4-methylcytosine sites using a deep learning approach with chemical properties

Waleed Alam[1], Hilal Tayara[2,*], Kil To Chong[1,3,*]

[1]Department of Electronics and Information Engineering, Jeonbuk National University, Jeonju 54896, South Korea; waleedtkr@jbnu.ac.kr
[2]School of International Engineering and Science, Jeonbuk National University, Jeonju, 54896, South Korea; hilaltayara@jbnu.ac.kr
[3]Advanced Electronics and Information Research Center, Jeonbuk National University, Jeonju 54896, South Korea; kitchong@jbnu.ac.kr
* Correspondence: hilaltayara@jbnu.ac.kr; kitchong@jbnu.ac.kr

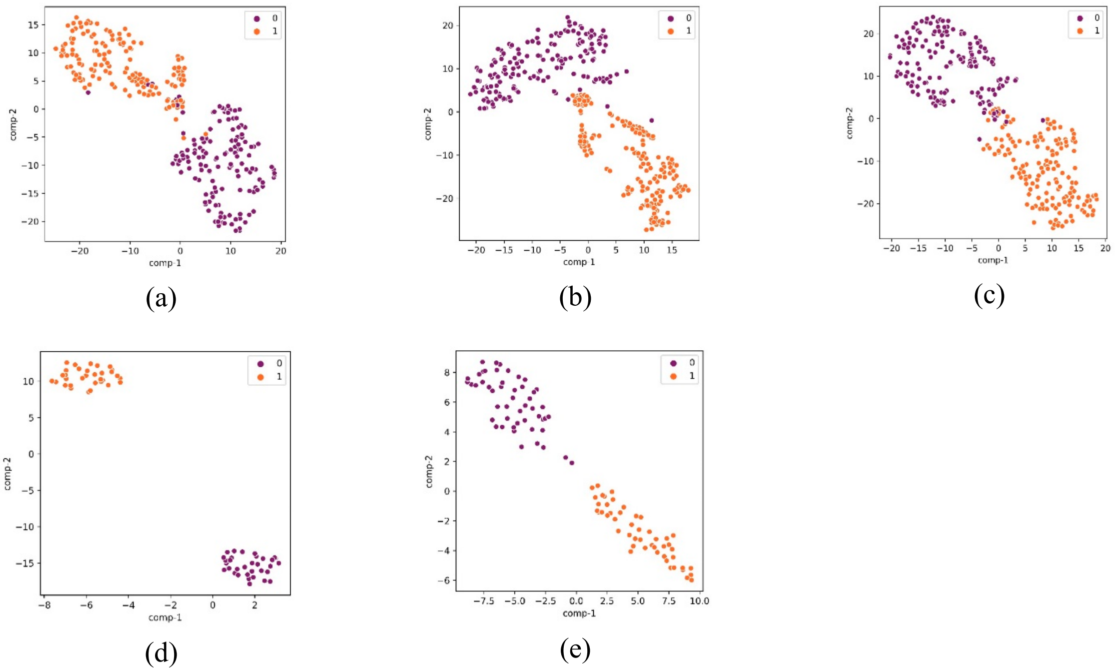

Figures S1. The t-SNE visualization of the learned features for *C. elegans* (a), *A. thaliana* (b), *D. melanogaster* (c), *E. coli* (d), and *G. pickeringii* (e). The '0' represents the features of the negative samples and '1' represents the features of the positive samples.

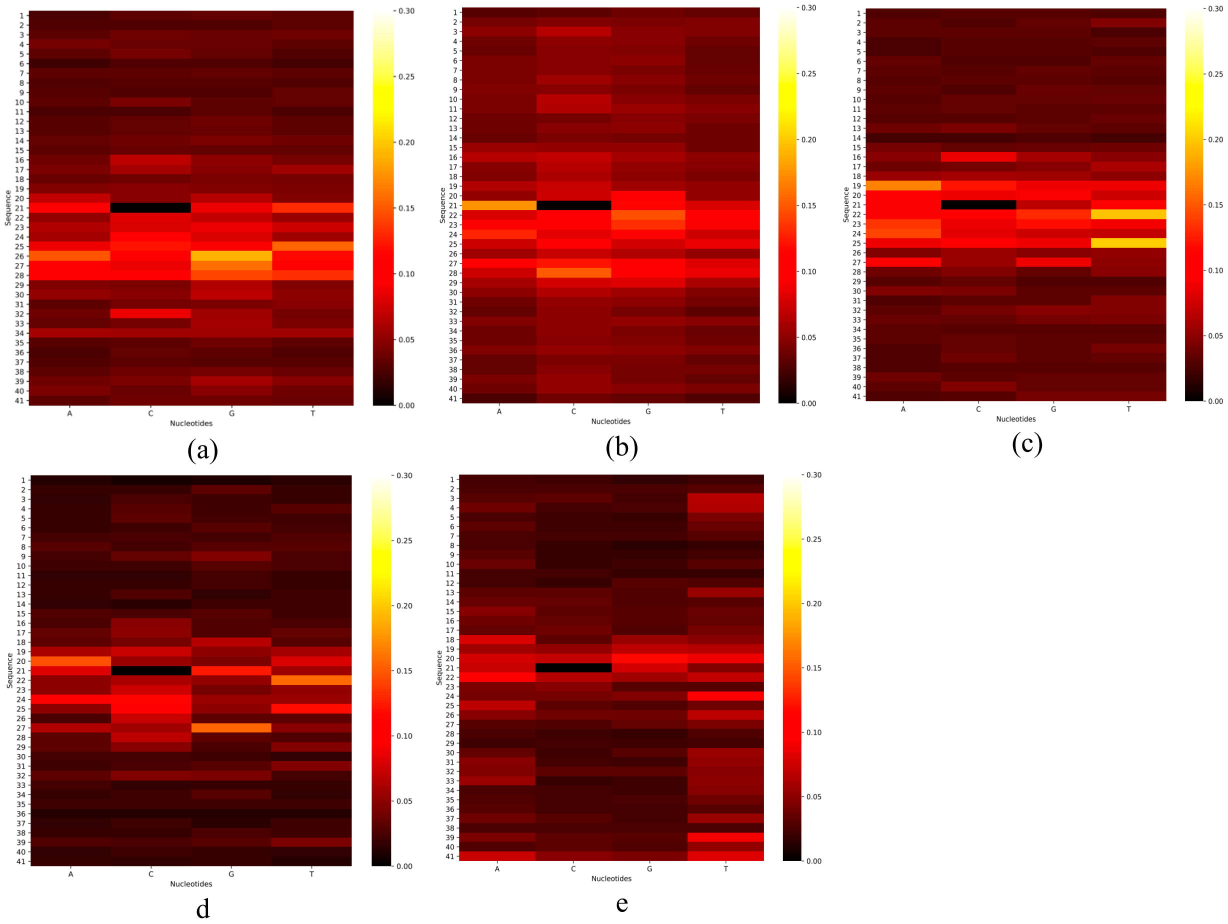

Figures S2. Heatmaps of in silico mutagenesis analysis for *C. elegans* (a), *A. thaliana* (b), *D. melanogaster* (c), *E. coli* (d), and *G. pickeringii* (e).

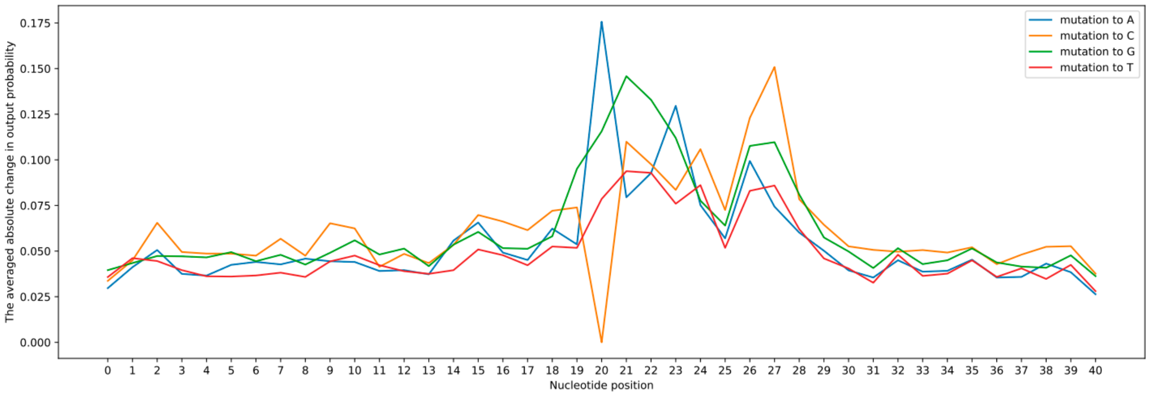

Figure S3. The effects of mutation on the prediction result in *A. thaliana*. It can be seen that mutations in the flanking regions, positions 0 to 18 and positions 28 to 40, have a small impact on the prediction performance. However, some of the mutations at positions 19 to 27 alter the prediction by more than 10%. The most noticeable alteration in the prediction occurs due to the mutations to Adenine (A) at position 20, and to to Cytosine (C) at position 27, more than 15%.

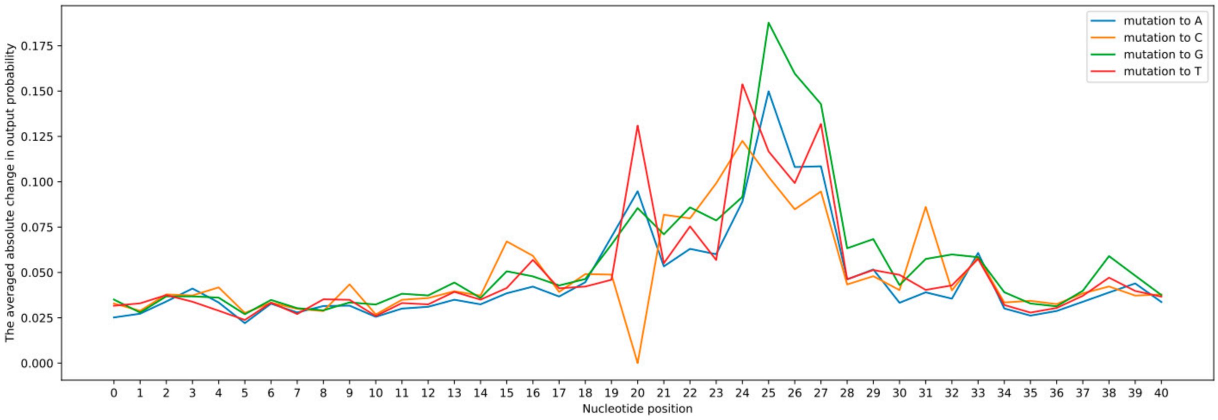

Figure S4. The effects of mutation on the prediction result in *C. elegans*. It can be seen that mutations in the flanking regions, positions 0 to 19 and positions 28 to 40, have a small impact on the prediction performance. However, some of the mutations at positions 20 to 27 alter the prediction by more than 10%. The most noticeable alteration in the prediction occurs due to the mutation to Guanine (G) at position 25, more than 17%.

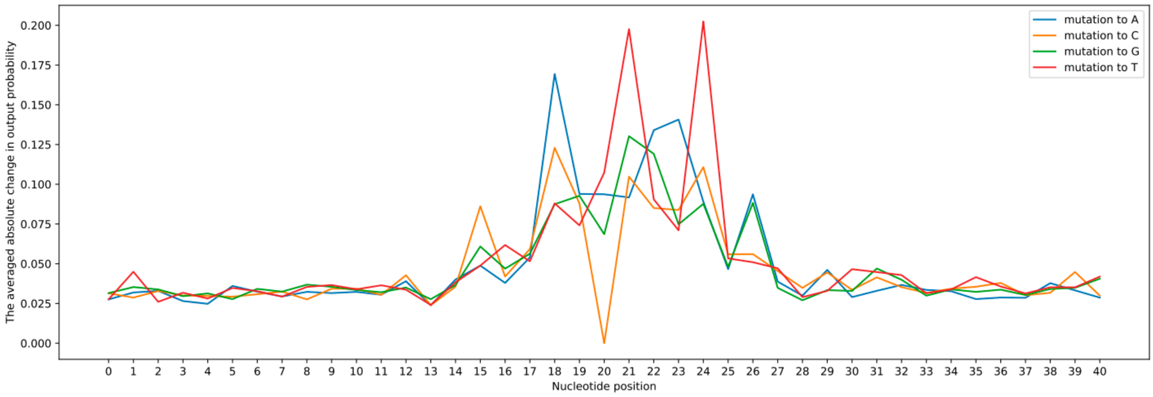

Figure S5. The effects of mutation on the prediction result in *D. melanogaster*. It can be seen that mutations in the flanking regions, positions 0 to 17 and positions 25 to 40, have a small impact on the prediction performance. However, some of the mutations at positions 18 to 24 alter the prediction by more than 10%. The most noticeable alteration in the prediction occurs due to the mutations to Thymine (T) at positions 21 and 24, and to Adenine (A) at position 18, more than 15%.

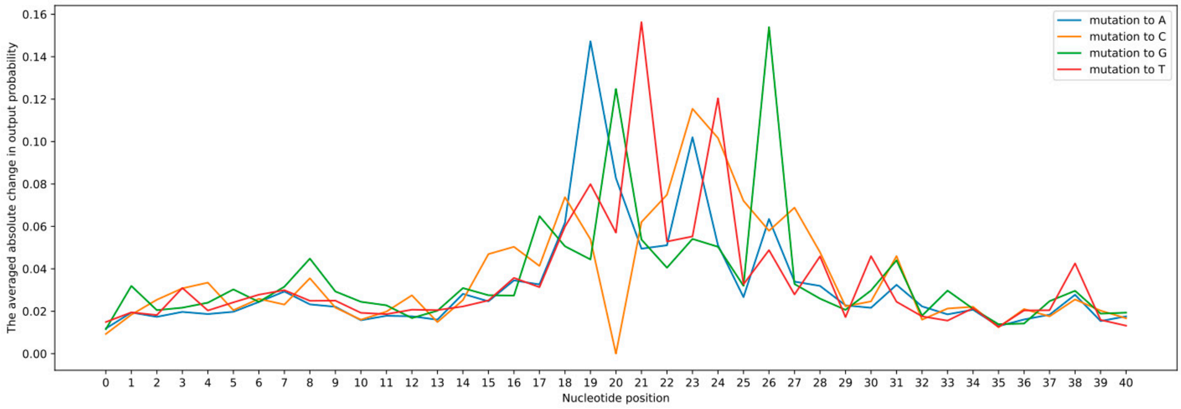

Figure S6. The effects of mutation on the prediction result in *E. coli*. It can be seen that mutations in the flanking regions, positions 0 to 18 and positions 27 to 40, have a small impact on the prediction performance. However, some of the mutations at positions 19 to 26 alter the prediction by more than 10%. The most noticeable alteration in the prediction occurs due to the mutations to Thymine (T) at position 21, and to Guanine (G) at position 26, more than 15%.

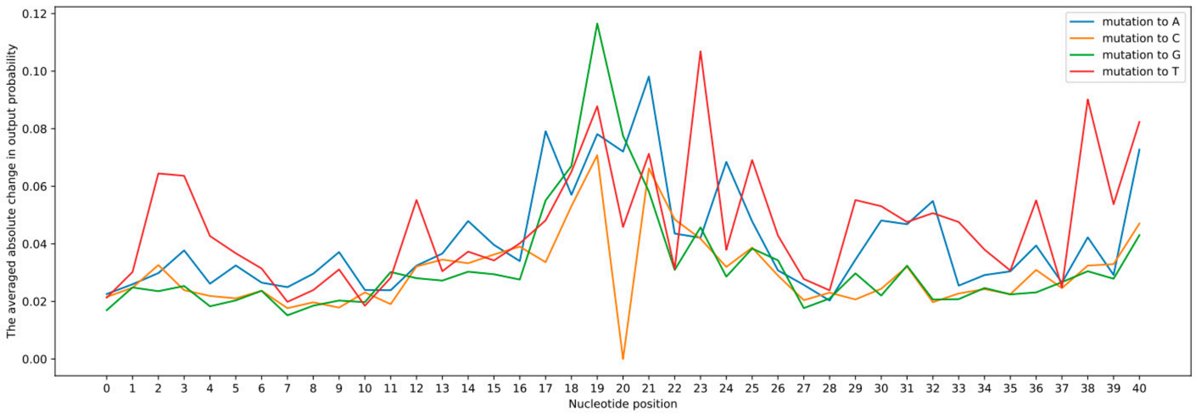

Figure S7. The effects of mutation on the prediction result in *G. pickeringii*. It can be seen that mutations to Guanine (G) at positions 19 and to Thymine (T) at position 23 alter the prediction by more than 10%. It can be noticed that the in silico analysis of *G. pickeringii* shows a different pattern compared with the other species in this study.

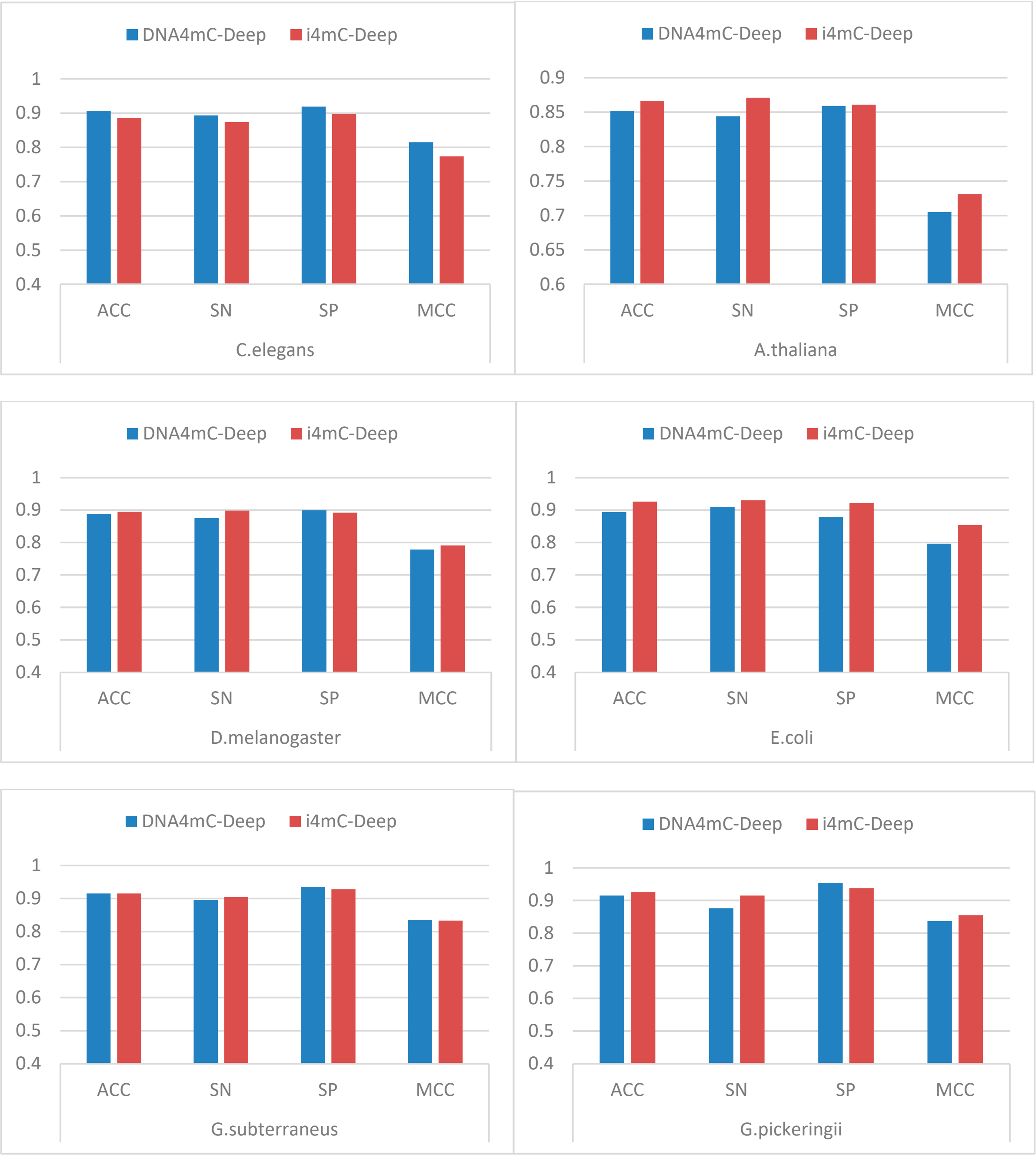

Figure S8. The comparison results between i4mC-Deep and DNA4mC-Deep after training the DNA4mC-Deep model on the six species in this study.

Table S1. The performance comparison between the i4mC-Deep and the DNA4mC-Deep after training the DNA4mC-Deep model on six species.

Dataset	Methods	ACC	SN	SP	MCC
C. elegans	DNA4mC-Deep	0.906	0.893	0.919	0.815
	i4mC-Deep	0.886	0.874	0.898	0.774
D. melanogaster	DNA4mC-Deep	0.888	0.876	0.899	0.778
	i4mC-Deep	**0.895**	**0.898**	**0.892**	**0.791**
A. thaliana	DNA4mC-Deep	0.852	0.844	0.859	0.705
	i4mC-Deep	**0.866**	**0.871**	**0.861**	**0.731**
E. coli	DNA4mC-Deep	0.894	0.91	0.879	0.796
	i4mC-Deep	**0.926**	**0.93**	**0.922**	**0.854**
G. subterraneus	DNA4mC-Deep	0.915	0.895	0.935	0.835
	i4mC-Deep	0.915	**0.904**	0.928	0.833
G. pinkeringii	DNA4mC-Deep	0.915	0.876	0.954	0.837
	i4mC-Deep	**0.926**	**0.915**	0.938	**0.855**

Table S2. The performance comparison between i4mC-Deep and pretrained cross-species model DNA4mC-Deep.

Dataset	Methods	ACC	SN	SP	MCC
C. elegans	DNA4mC-Deep	0.508	0.427	0.590	0.022
	i4mC-Deep	**0.886**	**0.874**	**0.898**	**0.774**
D. melanogaster	DNA4mC-Deep	0.497	0.320	0.675	-0.004
	i4mC-Deep	**0.895**	**0.898**	**0.892**	**0.791**
A. thaliana	DNA4mC-Deep	0.504	0.550	0.458	0.010
	i4mC-Deep	**0.866**	**0.871**	**0.861**	**0.731**
E. coli	DNA4mC-Deep	0.514	0.648	0.379	0.057
	i4mC-Deep	**0.926**	**0.93**	**0.922**	**0.854**
G. subterraneus	DNA4mC-Deep	0.524	0.679	0.370	0.060
	i4mC-Deep	**0.915**	**0.904**	**0.928**	**0.833**
G. pinkeringii	DNA4mC-Deep	0.449	0.370	0.527	-0.121
	i4mC-Deep	**0.926**	**0.915**	**0.938**	**0.855**
